# Anesthesia Management for Epicardial Pacemaker Electrode Implantation in a Patient With a History of Fontan Procedure: A Case Report

**DOI:** 10.7759/cureus.76407

**Published:** 2024-12-26

**Authors:** Tokimitsu Hibino, Yusuke Okui, Yoshie Toba

**Affiliations:** 1 Department of Anaesthesiology, Seirei Hamamatsu General Hospital, Hamamatsu, JPN

**Keywords:** fontan, lateral thoracotomy, one-lung ventilation (olv), pacemaker electrode implantation, post-fontan syndrome, sick sinus syndrome

## Abstract

One-lung ventilation is commonly used in lateral open chest surgery; however, it can increase pulmonary vascular resistance, which negatively affects Fontan circulation. Nevertheless, one-lung ventilation has a positive indication in post-Fontan patients. It allows surgery with lateral minimally invasive thoracotomy, which does not require a median sternotomy. Post-Fontan patients often have strong adhesions around the sternum and mediastinum due to multiple surgeries. Even worse, the large vessels sometimes adhere to the sternum, and a median sternotomy risks major hemorrhage. Since such risks can be avoided, one-lung ventilation is beneficial. Herein, we report our experience of one-lung ventilation anesthesia management for a post-Fontan patient who underwent pacemaker electrode implantation by right-sided minimally invasive thoracotomy. The Fontan circulation has a low tolerance for hypoxemia, so immediate treatment is necessary if hypoxemia develops during one-lung ventilation. Therefore, we connected the dependent lung side of the double-lumen tube to the anesthesia circuit and the nondependent lung side to the Jackson-Rees circuit, thereby completely separating the dependent and nondependent lungs. The Jackson-Rees circuit is highly versatile because the valve can be set to open and close freely, allowing the valve to be opened completely to administer oxygen, semi-closed to apply continuous positive airway pressure to the nondependent lungs, or ventilate the nondependent lungs at any desired time. We used this circuit to address hypoxemia during one-lung ventilation. Upon initiating one-lung ventilation, central venous pressure (CVP) increased from 8 to 19 mmHg, and SpO_2_ dropped from 99% to 83%. However, administering oxygen to the non-ventilated lung improved SpO2 to 98% and decreased CVP to 14 mmHg. Throughout the procedure, intermittent ventilation of the nondependent lung was performed cautiously to avoid disrupting the surgical field, allowing the operation to be completed safely. Intermittent ventilation of the nondependent lung using the Jackson-Rees circuit, without interfering with the operative field, was effective in maintaining oxygenation during one-lung ventilation in a patient with a history of Fontan procedure.

## Introduction

**Figure 1:**
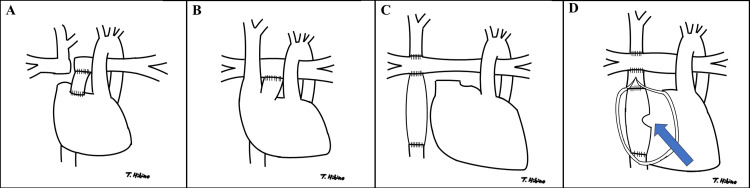
The types of Fontan procedure A: The original Fontan procedure. In this method, the superior vena cava is anastomosed end-to-end to the right pulmonary artery, and the right atrial appendage is anastomosed to the left pulmonary artery to close the atrial septal defect. B: Atrio-pulmonary connection. The right atrial appendage is anastomosed to the pulmonary artery, while the venae cavae remain unaltered. C: The epicardial conduit method. The superior vena cava is anastomosed directly to the pulmonary artery, and the inferior vena cava (IVC) is connected to the pulmonary artery via an artificial blood vessel through the extra-heart. D: The lateral tunnel method. The superior vena cava is anastomosed directly to the pulmonary artery. The IVC is anastomosed to the pulmonary artery via an artificial blood vessel that passes through the atrium. The arrow indicates the fenestration, a hole created to bypass the lungs when pulmonary vascular resistance rises, impairing pulmonary circulation. The fenestration allows blood to return to the atrium, maintaining cardiac output under high resistance conditions. However, this also causes venous blood from the IVC to enter the ventricle, reducing SpO_2_. The decision to create a fenestration depends on the patient’s condition and may also be made postoperatively if necessary.

The Fontan procedure is performed for complex cardiac malformations that cannot be repaired by biventricular surgery. It was first described by Fontan et al. in 1971 for tricuspid atresia [1]. In the original method described by Fontan et al., the superior vena cava was anastomosed end-to-end to the right pulmonary artery, and the right atrial appendage was anastomosed to the left pulmonary artery to close the atrial septal defect, allowing blood from the inferior vena cava to flow into the left pulmonary artery (Figure 1A) [1]. Although no major changes were made to the technique for about 10 years after its introduction, the atrio-pulmonary connection (Figure 1B) was developed. Currently, the epicardial conduit (Figure 1C) and the lateral tunnel methods (Figure 1D) are the most commonly performed procedures [2]. These latter two techniques are collectively referred to as total cavopulmonary connection (TCPC) because the superior and inferior venae cavae are anastomosed directly to the pulmonary artery. In all four of these procedures, the ventricle is no longer responsible for pulmonary circulation. Blood flows from the venae cavae through the pulmonary artery, becomes oxygenated in the lungs, returns to the atria, and is then pumped by the ventricles into the systemic circulation (Figure 1). Sufficient preload is necessary for the ventricles to eject an adequate stroke volume, and blood passing through the lungs defines the heart’s preload. Therefore, adequate pulmonary circulation is essential to maintain cardiac output. Since there is no pump, the pulmonary circulation requires an upstream and downstream pressure gradient to flow. This means high central venous pressure and low pulmonary vascular resistance (PVR) are needed, and increased PVR is detrimental to the Fontan circulation [2,3].

There are two main causes of increased PVR: constriction of pulmonary vessels in response to hypoxia, known as hypoxic pulmonary vasoconstriction (HPV), and compression of small pulmonary vessels during alveolar expansion [4]. Therefore, when managing anesthesia, the priority is to prevent hypercapnia and hypoxemia and to use regional anesthesia as much as possible to maintain spontaneous respiration (avoiding positive pressure ventilation) [5].

Patients who have undergone the Fontan procedure may develop post-Fontan syndrome, which includes arrhythmia, thromboembolism, protein-losing enteropathy, heart failure, pulmonary hypertension, liver cirrhosis, and renal failure over time [6]. Arrhythmias and pulmonary hypertension require aggressive treatment, as these complications directly impair cardiac output in post-Fontan patients. Bradyarrhythmias often require pacemaker implantation [7]. Regarding anatomical challenges in post-Fontan patients, transvenous pacemaker lead placement in the atrioventricular chambers is difficult. Consequently, pacemaker electrodes are typically implanted in the epicardium [8]. However, these patients often have undergone multiple thoracotomies [9], leading to significant adhesions in the mediastinum and pericardial sacs, making median sternotomy a high-risk option.

One way to avoid a median sternotomy is to approach the heart through a lateral open chest. To perform pacemaker electrode implantation, only the portion of the heart where the electrode is to be implanted needs to be exposed. A lateral thoracotomy without a median sternotomy and approach to the pericardial sac provides a minimally invasive route to access the heart with less adherent tissue. However, this approach requires one-lung ventilation (OLV) because the respiratory motion of the lung interferes with the operation.

Although OLV is commonly used in thoracic surgery, it can cause hypoxemia and HPV, which increases PVR. Additionally, the dependent lung must breathe at a higher ventilation volume than during bilobar ventilation, placing higher positive pressure on the alveoli of the dependent lung and further increasing PVR. This rise in PVR associated with OLV is detrimental to the Fontan circulation [10]. In this case, anesthesia with OLV was administered during a right-sided minimally invasive thoracotomy for pacemaker electrode implantation in a patient with sick sinus syndrome who had previously undergone the Fontan procedure. This report discusses the circulatory changes observed during OLV in a patient with Fontan circulation and introduces a new method for managing hypoxemia during OLV.

## Case presentation

A 28-year-old woman (weight: 61 kg; body mass index: 23.8 kg/m²; body surface area: 1.65 m²) with bradyarrhythmia was scheduled for pacemaker electrode implantation. At two months of age, she had undergone pulmonary artery banding and Blalock-Taussig shunt creation via left-lateral thoracotomy, which is why the present approach was a right-lateral thoracotomy. At 20 months of age, she underwent the Fontan procedure (atriopulmonary connection) (Figure 1B) to treat tricuspid atresia. Due to paroxysmal supraventricular tachycardia, she underwent atrial ablation and a TCPC conversion (without fenestration) at age 25. Subsequently, she developed bradycardia and was diagnosed with sick sinus syndrome. As bradycardia progresses, atrial filling increases, and pulmonary venous pressure rises. Consequently, pulmonary blood flow in the Fontan circulation becomes difficult to maintain. Her preoperative hepatic venous blood flow velocity measurement by abdominal echocardiography often showed reflux waves. Additionally, her paroxysmal supraventricular tachycardia occasionally recurred due to impaired sinus node and atrial function. In this case, atrial pacing electrodes were to be implanted to prevent bradycardia and intra-atrial reentrant tachycardia. In the TCPC Fontan circulation, the venae cavae are not connected to the atria, making transvenous implantation of a pacing lead into the atria impossible (Figure 1C, D). We therefore decided to surgically implant a pacemaker electrode. There were strong adhesions to the sternum and mediastinum. Additionally, the ascending aorta and innominate vein were in contact with the dorsal surface of the sternum, making a median sternotomy a high-risk option due to the potential for major hemorrhage. Consequently, a right-sided minimally invasive thoracotomy was chosen for the procedure.

The patient had been diagnosed with cirrhosis, a late complication of Fontan circulation, at age 20, with a preoperative Child-Pugh classification of B (score of 8) (Table 1). This diagnosis was primarily based on abdominal echographic findings. A liver biopsy was not performed due to the risk of hemorrhage associated with elevated hepatic venous pressure. Although our department performs paravertebral blocks for postoperative pain relief in similar surgeries, the block was not performed in this case because the prothrombin time was prolonged. At our institution, some patients who have undergone Fontan procedures experience delayed awakening from total intravenous anesthesia, with one such case being diagnosed with postoperative liver dysfunction [11]. In this case, sevoflurane was used for anesthesia maintenance to avoid intravenous anesthetics metabolized by the liver. Desflurane was not used because of its potential to increase PVR [12]. However, midazolam and ketamine were used during induction. Ketamine increases systemic vascular resistance and prevents hypotension during anesthesia induction. Our institution often uses midazolam and ketamine for anesthesia induction in patients with low cardiac function.

**Table 1:** Child-Pugh classification Child-Pugh Class A is defined as a total score of 5 to 6, Class B as a total score of 7 to 9, and Class C as a total score of 10 to 15. The three left-hand columns of the table show the components of each score in the Child-Pugh classification. The two columns on the right side of the table show the patient’s laboratory values and other components with their corresponding scores. With a total score of 8, this patient was classified as Child-Pugh Class B. Hepatic dysfunction had already been observed in this case. Additionally, other anesthesia managements of post-Fontan patients at our institution had shown delayed awakening and postoperative hepatic dysfunction. Therefore, we decided to avoid anesthetics with hepatic metabolism as much as possible, selecting sevoflurane for this patient. *Mild hepatic encephalopathy: Equivalent to hepatic encephalopathy stages I and II. Stage I is characterized by euphoria, reversal of day-night rhythm, and sluggishness. Stage II is characterized by disorientation and asterixis. **Mild ascites is defined as inferred ascitic fluid retention greater than 1 L. Moderate or greater ascites is defined as estimated ascitic fluid retention greater than 3 L. PTINR: prothrombin time-international normalized ratio.

Score	1	2	3	Our case	Points
Hepatic encephalopathy*	None	Mild	Coma	None	1
Ascites**	None	Mild	>Moderate	Mild	2
Serum bilirubin (mg/dL)	<2.0	2.0-3.0	>3.0	1.0	1
Serum albumin (g/dL)	>3.5	2.8-3.5	<2.8	3.6	1
PTINR	<1.7	1.7-2.3	>2.3	2.46	3
Total					8

Preoperative cardiac catheterization showed a mean pulmonary artery pressure of 14 mmHg and a PVR of just 0.5 Wood units. This PVR was low enough in the Fontan circulation to be ideal. Nevertheless, the patient complained of ascites and anorexia. Additionally, approximately 40 days prior to this surgery, she experienced diarrhea five to six times a day. These symptoms were thought to be due to increased venous pressure in the abdomen, with bradyarrhythmia suspected as an exacerbating factor.

**Figure 2:**
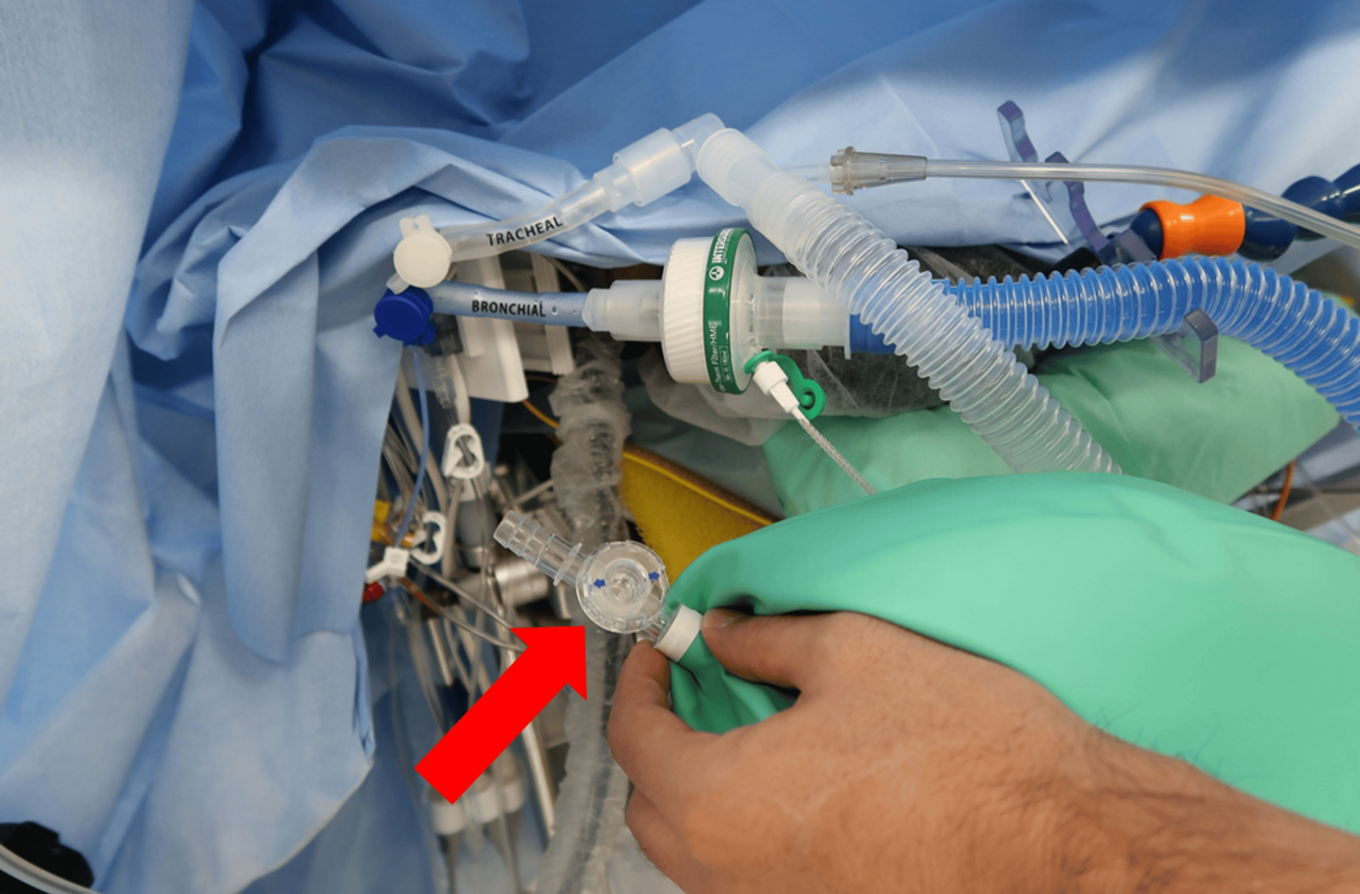
Anesthesia circuit The bronchial lumen of the ventilated lung was connected to the anesthesia circuit, while the tracheal lumen of the non-ventilated lung was connected to the Jackson-Rees circuit. The arrow indicates the valve of the circuit, which can freely adjust the degree of opening and closing, from fully open to fully closed. This setup allowed independent oxygen administration, CPAP application, and ventilation of the nondependent lung at any time, separate from the respiratory cycle of the anesthesia circuit. CPAP: continuous positive airway pressure

In the operating room, continuous monitoring included electrocardiography, arterial blood pressure, and SpO_2_ measurements. Vital signs, including heart rate, arterial blood pressure, SpO_2_, and central venous pressure (CVP) (after central venous catheter insertion), were recorded every three seconds. Baseline measurements showed a blood pressure of 130/60 mmHg, a heart rate of 54 beats/min, and an SpO_2_ of 92% on room air. The CVP is the driving pressure of the pulmonary artery flow in the Fontan circulation, and an elevated CVP indicates that pulmonary artery flow cannot be maintained at normal levels due to increased PVR. We carefully monitored for elevated CVP. Hypoxemia increases PVR, so maintaining a high SpO_2_ is important to support the Fontan circulation. Although blood from the venae cavae no longer enters the ventricle, venous blood from the coronary sinus continues to flow into the atrium, potentially lowering arterial blood oxygen saturation compared to healthy individuals. The baseline SpO_2_ in this case was 92%, so care was taken to keep it at least above this level. Anesthesia was induced with midazolam, ketamine, remifentanil, and rocuronium and maintained with sevoflurane and remifentanil. Dopamine and dobutamine were administered at the start of anesthesia to maintain cardiac output, while nitroglycerin and milrinone were given to keep PVR low. A 35Fr left-side double-lumen tube (Coopdech®, Daiken Medical Co. Ltd., Osaka, Japan) was intubated. After intubation, SpO_2_ increased to 99% with pure oxygen. The bronchial lumen to the ventilated lung was connected to the anesthesia circuit, while the tracheal lumen to the non-ventilated lung was connected to the Jackson-Rees circuit (tkb Criticare®, TOKIBO Co. Ltd., Tokyo, Japan) (Figure 2).

The reason we used the Jackson-Rees circuit is because of its versatility. As shown in Figure 2, this circuit has a valve. By turning the dial from fully released to fully closed, we can freely change the degree of valve opening. When the valve is completely released, the airway pressure on the nondependent lung side does not increase during oxygen administration, and the nondependent lung remains collapsed. When the valve is semi-closed, any pressure can be applied to the circuit, allowing for continuous positive airway pressure (CPAP) as well as oxygen administration. In addition, when the valve is semi-closed and the bag is pushed, ventilation can be provided.

In this method, the nondependent lung completely separates from the dependent lung and can be ventilated at different times from the dependent lung. The Jackson-Rees circuit can ventilate the nondependent lungs when the surgical procedure is stopped. Thus, the ventilation does not interfere with the surgical procedure and can deal with hypoxemia, which is detrimental to the Fontan circulation.

**Figure 3:**
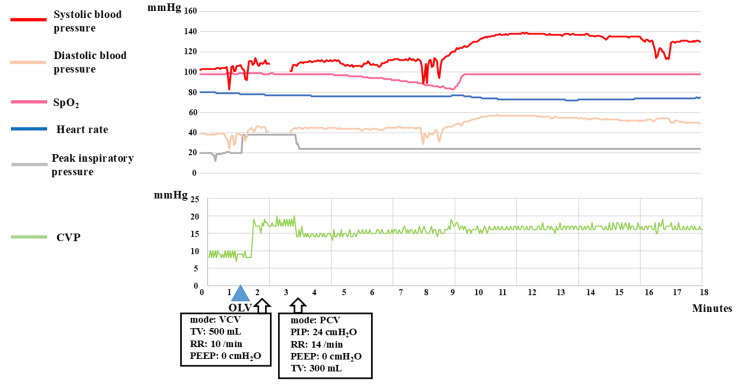
Circulatory changes during initiation of one-lung ventilation Blood pressure, heart rate, CVP, peak inspiratory pressure, and SpO2 were recorded at 3-second intervals for approximately 18 minutes, from the start of OLV to the beginning of surgery. Ventilation mode was switched to PCV 2 minutes after the start of OLV and remained unchanged until extubation. CVP: central venous pressure, OLV: one-lung ventilation, VCV: volume-controlled ventilation, PCV: pressure-controlled ventilation, TV: tidal volume, RR: respiratory rate, PEEP: positive end-expiratory pressure, PIP: peak inspiratory pressure.

Immediately after initiating the OLV, CVP increased from 8 to 19 mmHg, and blood pressure decreased from 103/37 mmHg to 93/32 mmHg (Figure 3). Within 9 seconds, blood pressure increased from 92/36 mmHg to 111/43 mmHg. Two minutes later, a high peak inspiratory pressure was detected, and the ventilation was switched from volume-controlled to pressure-controlled ventilation. Upon ventilator mode switch, peak inspiratory pressure decreased from 38 cmH₂O to 24 cmH₂O, and blood pressure immediately increased from 101/40 mmHg to 110/45 mmHg. CVP also quickly decreased from 19 mmHg to 14 mmHg.

The high airway pressure caused the peripheral pulmonary arteries around the alveoli to be compressed, resulting in an increase in PVR, which reduced the venous return from the pulmonary circulation to the heart and thus the blood pressure dropped. The decrease in airway pressure caused the peripheral pulmonary arteries to become decompressed, resulting in a decrease in CVP and an increase in blood pressure.

Three minutes after the start of OLV, SpO_2_ decreased from 99% to 83% over 5 minutes. CVP increased from 14 to 18 mmHg, and blood pressure fell from 111/45 to 89/29 mmHg. This change highlighted that hypoxemia-induced HPV increased PVR and the pulmonary circulation decreased in flow. After oxygen administration to the non-ventilated lung, CVP decreased to 15 mmHg, and blood pressure gradually improved (Figure 3).

During the operation, compression of the nonventilated lung made it difficult to maintain SpO_2_ above 95% with oxygen administration alone. Intermittent ventilation of the non-ventilated lung was performed when it did not interfere with the procedure. During the first half of the operation, oxygen was sent to the nondependent lungs by pushing the bag of the Jackson-Rees circuit at a pressure of about 20 cmH₂O about twice per minute. By the time the operative maneuver reached the pericardial sac, the nondependent lungs were compressed by the surgical instruments, and the SpO_2_ gradually decreased. The CVP also gradually increased to 20 mmHg. When the primary surgeon was operating in the surgical field, the valve of the Jackson-Rees circuit was released to administer oxygen, and when he stopped operating in the surgical field, such as changing surgical instruments, the valve was semi-closed for rapid ventilation (ventilation frequency was once every 10 to 20 seconds).

**Figure 4:**
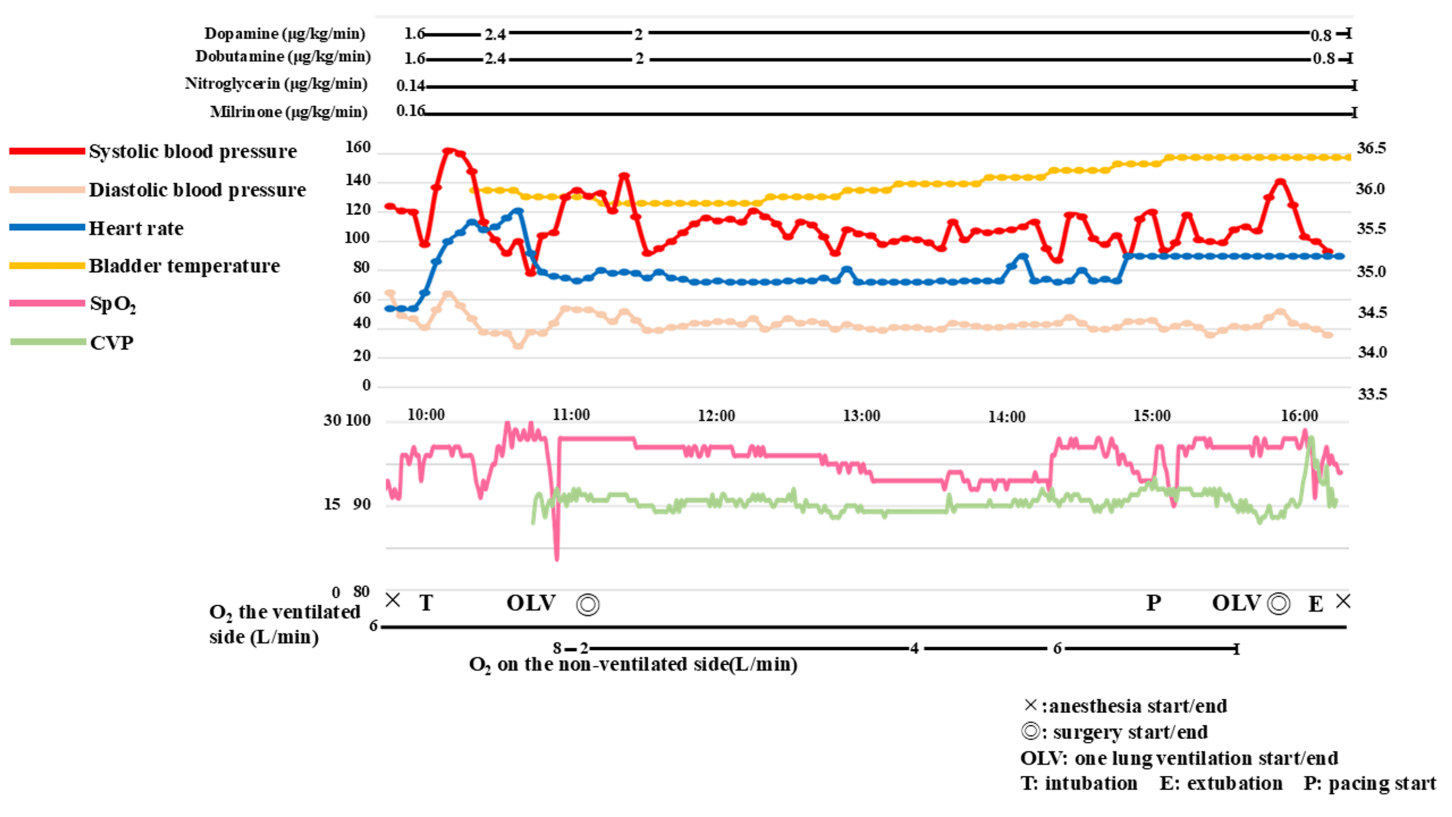
Anesthesia record The left axis of the chart shows the scales for blood pressure and heart rate, while the right axis displays the scale for bladder temperature. CVP: central venous pressure, OLV: one-lung ventilation.

Epicardial pacemaker electrodes were successfully implanted, and atrial pacing was initiated. At the start of pacing, CVP decreased from 20 to 13 mmHg (Figure 4). Measurement of hepatic venous blood flow velocity by transesophageal echocardiography (TEE) showed the disappearance of the systolic reflux phase. The blood flow velocity in the epicardial conduit had been turbulent, but after pacing, the turbulent component disappeared, and laminar flow was observed. These results suggested that atrial pacing improved atrioventricular synchrony. After a comprehensive cardiac evaluation with TEE (Table 2), the operation was concluded, and the patient was extubated and transferred to the intensive care unit in stable condition. CVP ranged from 9 to 12 mmHg until the central venous catheter was removed on postoperative day two. From postoperative day three, she had watery stools twice a day, which improved to soft stools once a day from postoperative day nine. Appetite also improved during the same period. Abdominal ultrasonography performed on postoperative day 40 showed a small amount of ascites at the hepatic margin, significantly less than that observed preoperatively. Abdominal ultrasonography performed 8 months after surgery showed that the ascites had disappeared completely.

**Table 2:** Transesophageal echocardiographic findings before and after the pacing initiation Before pacing, dopamine and dobutamine were administered at 2.4 μg/kg/min, and after initiating pacing, they were administered at 2.0 μg/kg/min. EF clearly improved despite the reduced dosage of the inotropic agents. TCPC conduit blood flow velocity significantly decreased, and the turbulent component disappeared after pacing. In this case, cardiac output was not measured before and after pacing. However, assuming cardiac output did not change significantly after pacing, a lower transpulmonary gradient would have been required to maintain pulmonary circulation, leading to a reduction in conduit blood flow velocity. E wave, A wave, lateral E/E', and pulmonary venous blood flow velocity were used to assess relaxation capacity. Heart rate was higher after the start of pacing, resulting in a relatively shorter relaxation time. Although this might have been expected to deteriorate parameters representing relaxation capacity, the measurements remained virtually unchanged. *The values for these reference ranges are cited from Mathew et al. [13]. ^†^The values for these reference ranges are cited from Cohen et al. [14]. Data are presented as mean value (confidence interval). ^‡^A velocity greater than 150 cm/s is suggestive of TCPC conduit obstruction. EF: ejection fraction, PVs: pulmonary venous flow systolic wave, PVd: pulmonary venous flow diastolic wave, PVa: pulmonary venous flow atrial reverse wave, TCPC: total cavopulmonary connection.

Measurement (units)	Before pacing	After initiating pacing	Reference range
Heart rate (beats/min)	76	90	
EF(%)*	55	65	53-73
Transmitral E wave (cm/s)^†^	73	70	72 (44-100)
Transmitral A wave (cm/s)^†^	44.5	40.6	40 (20-60)
Lateral E/E' ratio*	9.1	8.1	<13
PVs (cm/s)^†^	68.8	41	48 (30-66)
PVd (cm/s)^†^	116	110	50 (30-70)
PVa (cm/s)^†^	41.5	54	19 (11-27)
Flow velocity of TCPC conduit (cm/s)*	63.5	48.7	20-50^‡^

## Discussion

OLV is generally associated with hypoxemia due to increased intrapulmonary shunting caused by the collapse of the nondependent lung [15]. As the local partial pressure of alveolar oxygen decreases, HPV is activated, vascular smooth muscle in the pulmonary circulation contracts, and blood flow is redirected to the well-oxygenated, dependent lung [16]. The extent to which the dependent lung in post-Fontan patients can accommodate increased blood volume is uncertain. In some previous cases, large decreases in blood pressure requiring inotropes and vasopressors were observed after OLV initiation [10,17-19]. One patient developed marked tachycardia of 130-180 beats/min [17]. In another case, OLV was performed relatively safely [19]. These different responses to OLV may be the result of differences in reserve capacity to accommodate rapid changes in PVR. Eagle et al. noted that a sudden increase in PVR can lead to right-left shunting and baffle leakage via fenestration of the epicardial conduit [4].

In our previous case, we used cardiac catheterization to assess whether a patient who had undergone the Fontan procedure could tolerate pneumonectomy for lung cancer. A balloon occlusion test was performed on the left pulmonary artery. Although CVP remained unchanged before and after balloon occlusion, angiography revealed that some contrast agents flowed from the superior vena cava to the atrium through a previously nonfunctional fenestration [20]. This bypass flow suggested that the balloon occlusion allowed blood to escape through the fenestration, buffering the rise in CVP. This indicated that the pulmonary arterial vascular bed on the ventilated side was unable to accommodate the entire pulmonary blood flow. At the same time, the flow of blood through the epicardial conduit into the atrium via fenestration maintained preload and cardiac output despite the increase in PVR. This phenomenon is consistent with that noted by Eagle et al. They also mentioned that the increase in airway pressure on the dependent side of the lung during OLV initiation can increase PVR and interfere with post-Fontan pulmonary circulation [4].

In this case, immediately after initiation of OLV with volume-controlled ventilation, there was an increase in peak inspiratory pressure from 20 cmH₂O to 38 cmH₂O, an increase in CVP from 8 mmHg to 19 mmHg, and a decrease in blood pressure from 103/37 mmHg to 93/32 mmHg. Elevated airway pressure likely contributed to the increase in PVR. Elevated PVR due to high airway pressure can be prevented by optimizing ventilation conditions. In this case, the patient should have been set to pressure-controlled ventilation mode when OLV was started. Without a fenestration to buffer the elevated CVP, CVP increased immediately. The drop in blood pressure was probably due to reduced venous return. As in our previous case, described above, if fenestration was present, even if the pulmonary circulation was impaired due to increased PVR, blood would bypass the lungs through the fenestration and return to the heart, preventing a sudden decrease in blood pressure. In this case, there was no fenestration, and the increased PVR, decreased flow in the pulmonary circulation, and decreased preload to the heart likely resulted in a sudden drop in blood pressure. However, our anesthesia monitoring system records vital signs every three seconds, and the data showed that while blood pressure initially dropped with the rise in CVP, it recovered within nine seconds, long before ventilatory adjustments could reduce CVP. During the first 20 minutes of OLV, the heart rate remained almost unchanged. Therefore, a compensatory mechanism of cardiac output via the autonomic nervous system was unlikely to be active. Similarly, inotropic dose escalation was unlikely to have caused recovery from hypotension just after OLV initiation, as the inotropic dose had been increased as much as 25 minutes before the start of OLV, and its effect was unlikely to have appeared at a time after the start of OLV.

Eagle et al. stated that maintaining an optimal transpulmonary gradient achieves adequate pulmonary blood flow and cardiac output [4]. Supposing the pulmonary artery diameter remains unchanged, the transpulmonary pressure increases as PVR rises, CVP rises, and the transpulmonary gradient is maintained. For the same transpulmonary gradient with the same vessel diameter, pulmonary circulation flow can be maintained. Thus, the increase in PVR can be compensated for by the increase in CVP. If the diameter of the peripheral pulmonary artery contracts, depending on its diameter, a higher transpulmonary gradient than usual may be required to maintain pulmonary circulation. How much transpulmonary gradient is required to maintain pulmonary circulation during an increase in PVR may depend on the original flow capacity of the pulmonary artery. Therefore, the lower the original PVR, the greater the reserve capacity for situations in which PVR rises rapidly, such as OLV. In this case, the baseline PVR was sufficiently low, and the increased transpulmonary gradient could have preserved venous return volume as CVP rose. Additionally, when SpO_2_ decreased and CVP increased later during the procedure, blood pressure rose rather than fell, suggesting that the increase in CVP did not directly lead to a reduction in venous return or cardiac output.

In this case, the airway pressure should be kept as low as possible because a sudden increase in airway pressure can disrupt the Fontan circulation. When starting OLV, volume-controlled ventilation should be switched to pressure-controlled ventilation immediately.

Regarding ventilator settings, positive end-expiratory pressure (PEEP) would have increased the maximal airway pressure; therefore, PEEP was not applied in this case.

In Fontan patients, hepatic venous congestion becomes problematic when CVP exceeds 15 mmHg, and mortality increases when CVP exceeds 20 mmHg [3]. Thus, CVP is the most important monitor in the anesthetic management of Fontan patients. SpO_2_, EtCO_2_, and airway pressure are also monitored as they may increase or decrease CVP, and changes in these factors should be addressed aggressively. Changes in CVP induce changes in blood pressure. Hypotension can cause a drop in coronary perfusion pressure and a decrease in venous pressure in the Fontan circulation, risking disruption of the Fontan circulation. Therefore, hypotension must be carefully monitored and treated immediately.

Arrhythmias in patients with a history of Fontan procedure can reduce cardiac efficiency and worsen heart failure in the long term [21]. Pacemaker implantation is a treatment option for bradyarrhythmias [22]. In this patient, cardiac function was assessed by TEE before surgery (heart rate: 73 beats/min under dopamine 2.4 μg/kg/min and dobutamine 2.4 μg/kg/min) and after pacing began (pacing at AAI 90 beats/min under dopamine 2 μg/kg/min and dobutamine 2 μg/kg/min) (Table 2). Each parameter shown in Table 2 can vary with heart rate. Ejection fraction (EF) is considered a load-dependent index, and mitral inflow velocity and pulmonary vein velocity are influenced by heart rate. In fact, these waveforms fuse as the heart rate increases [13]. Hence, when the heart rate changes, comparison and evaluation of echocardiographic values are challenging. Although it is difficult to accurately assess echographic findings due to changes in heart rate, a gradual CVP decrease was observed from the start of pacing. This decrease in CVP may have resulted in a decrease in TCPC conduit blood flow velocity. Additionally, the disappearance of the retrograde component of blood flow in the TCPC conduit, which was present before pacing, might have contributed to the lower CVP and subsequent improvement in clinical symptoms. Despite the reduced catecholamine dose, TEE findings, especially EF, suggested improved cardiac contractility. Postoperatively, the patient showed improvements in food intolerance, resolution of diarrhea and intestinal edema, and disappearance of ascites, suggesting a reduction in venous pressure.

In this case, EF, CVP, and other parameters improved with the start of pacing. These results are encouraging for post-Fontan patients suffering from heart failure. However, these data are from a single case and cannot be directly extrapolated to other post-Fontan patients. Further data are needed to compare cardiac function before and after pacing therapy. Pacemaker implantation may contribute to improved cardiac function in patients with post-Fontan syndrome and could be considered for post-Fontan patients in the future [23]. Anesthetic management of post-Fontan patients undergoing isolated lung ventilation may improve the success rates of pacemaker implantation, as a lateral open chest approach can manage strong adhesions and access the pericardial sac.

## Conclusions

Pacing electrodes were inserted for a bradyarrhythmia in a patient with Fontan circulation. Since the sternum was attached to the innominate vein and ascending aorta, a median open chest approach was considered dangerous, so a right-sided open chest approach was chosen. The right lung hid the operative field because of the lateral thoracotomy, so OLV was necessary. Low PVR is important to maintain Fontan circulation, and OLV increases PVR. In this case, oxygenation to the nondependent lung by the Jackson-Rees circuit was effective in dealing with hypoxemia during OLV. The Jackson-Rees circuit can open and close valves freely. Thus, it is a highly versatile ventilation system that can be used simply to administer oxygen, to administer oxygen while applying CPAP, or to ventilate at any time by pushing the bag by hand. Since the Fontan circulation is less tolerant of hypoxemia, the Jackson-Rees circuit may be a best practice for the management of the nondependent lung during OLV.

In this patient, CVP decreased as pacing was initiated, and EF improved. Furthermore, postoperatively, the complete disappearance of ascites suggested improvement of intra-abdominal venous hypertension. The significant improvement in these clinical conditions began after pacing. Although this is a case report and cannot be extrapolated to all post-Fontan patients, it may offer hope to those suffering from post-Fontan syndrome. In the future, prospective multicenter cohort studies should be conducted to investigate the effects of pacing on arrhythmia control and cardiac resynchronization therapy in post-Fontan patients.
